# Altered Anatomical Network in Early Blindness Revealed by Diffusion Tensor Tractography

**DOI:** 10.1371/journal.pone.0007228

**Published:** 2009-09-28

**Authors:** Ni Shu, Yong Liu, Jun Li, Yonghui Li, Chunshui Yu, Tianzi Jiang

**Affiliations:** 1 LIAMA Center for Computational Medicine, National Laboratory of Pattern Recognition, Institute of Automation, Chinese Academy of Sciences, Beijing, People's Republic of China; 2 Department of Radiology, Xuanwu Hospital of Capital Medical University, Beijing, People's Republic of China; 3 State Key Laboratory of Cognitive Neuroscience and Learning, Beijing Normal University, Beijing, People's Republic of China; Indiana University, United States of America

## Abstract

The topological architecture of the cerebral anatomical network reflects the structural organization of the human brain. Recently, topological measures based on graph theory have provided new approaches for quantifying large-scale anatomical networks. Diffusion MRI studies have revealed the efficient small-world properties and modular structure of the anatomical network in normal subjects. However, no previous study has used diffusion MRI to reveal changes in the brain anatomical network in early blindness. Here, we utilized diffusion tensor imaging to construct binary anatomical networks for 17 early blind subjects and 17 age- and gender-matched sighted controls. We established the existence of structural connections between any pair of the 90 cortical and sub-cortical regions using deterministic tractography. Compared with controls, early blind subjects showed a decreased degree of connectivity, a reduced global efficiency, and an increased characteristic path length in their brain anatomical network, especially in the visual cortex. Moreover, we revealed some regions with motor or somatosensory function have increased connections with other brain regions in the early blind, which suggested experience-dependent compensatory plasticity. This study is the first to show alterations in the topological properties of the anatomical network in early blindness. From the results, we suggest that analyzing the brain's anatomical network obtained using diffusion MRI data provides new insights into the understanding of the brain's re-organization in the specific population with early visual deprivation.

## Introduction

The human brain is a complex natural system, which is capable of carrying out complicated behaviors. However, scientists know little about the neurobiological mechanisms behind higher-level brain functions. In recent years, several researchers have proposed network models and graph theory based topological measures as tools for increasing our understanding of the structural organization and functional mechanisms of the brain [1]–[10]. Recent studies that employed network models have revealed that brain networks exhibit small-world attributes [2], [6]–[13] and modular structure [14]–[16]. These findings support the view that the human brain has evolved a complex, but efficient, neural architecture to maximize the power of information processing [2], [17].

The cerebral anatomical network, which characterizes the global architecture of the anatomical connection pattern in the human brain, is critically important for understanding the underlying structural substrate of brain functions and can be expected to provide new insights into the ways that brain function is affected in certain disease states [4], [10]. To construct anatomical networks for various mammalian species, researchers have used axonal tracing methods to chart macroscopic connection patterns in the cerebral cortex [18]–[22]. However, the invasive nature of the procedure makes it an unlikely candidate for human connectivity analyses in vivo. Recently, noninvasive imaging methods, diffusion tensor imaging (DTI) coupled with fiber tracking algorithms, have come to serve as a feasible alternative to the tracer methods. By measuring the in vivo movement of water molecules within the brain tissues, DTI provides estimates of the direction of a local fiber bundle at each voxel [23]–[25]. Researchers can then infer inter-regional anatomical connections from local estimates using diffusion tensor tractography (DTT) [26]–[30].

Recently, several studies have constructed brain anatomical networks using diffusion MRI in healthy populations [9], [11], [13], [15], [31]. By calculating the anatomical connection probabilities between any two regions, some of these have modeled the brain as a non-directed weighted network [11], [31]. Similarly, Gong et al. constructed a binary anatomical network which used the existence/absence of regional connections [13], based on deterministic DTT techniques. In both cases the researchers employed the automated anatomical labeling (AAL) template [32] to parcellate brain regions. Moreover, to overcome the problem of fiber crossing in DTI, a diffusion spectral imaging (DSI) study suggested a method for constructing a high-resolution connection matrix for each subject with thousands of nodes defined at a voxel population level rather than a regional level [9]. Although the various studies proposed different strategies to define the nodes and connections of the anatomical network, all of them investigated normal people, and all revealed that the cortical networks of the human brain have a “small-world” topology, which is characterized by large clustering coefficient and short average path length [2], [33]. However, no diffusion MRI study has investigated changes in the brain anatomical network in early blind subjects, who lost sight at birth or within the first year of age (Table 1).

**Table 1:** Demographic data of the early blind subjects.

Case number	Gender	Age, years	Age onset, years	Causes of blindness	Massage	Piano or guitar practicing
1	Male	20.9	0	Retinitis pigmentosa	Yes	Yes
2	Male	24.6	0	Optic nerve atrophy	Yes	No
3	Male	19.1	0	Retinitis pigmentosa	No	Yes
4	Male	24.6	0	Retinitis pigmentosa	Yes	No
5	Male	22.4	<1	Congenital glaucoma	Yes	No
6	Male	29.3	0	Optic nerve hypoplasia	No	Yes
7	Male	23.4	<1	Congenital glaucoma	Yes	No
8	Male	20.8	<1	Congenital glaucoma	Yes	No
9	Male	18.7	0	Optic nerve hypoplasia	Yes	Yes
10	Male	19.0	0	Retrolental fibroplasia	Yes	Yes
11	Female	15.6	0	Optic nerve atrophy	No	No
12	Female	18.4	0	Retinitis pigmentosa	No	No
13	Female	21.7	0	Congenital glaucoma	Yes	Yes
14	Female	22.8	0	Retinitis pigmentosa	Yes	No
15	Female	27.7	0	Optic nerve atrophy	Yes	Yes
16	Female	22.7	0	Congenital cataract	No	No
17	Female	24.9	0	Optic nerve hypoplasia	Yes	No

Note: “Yes” in the massage column refers to a blind has learned or engaged in massage for more than 1 year; “No” in the massage column refers to a blind has learned or engaged in massage for less than 1 year or has never learned massage; “Yes” in the piano or guitar practicing column refers to a blind has practiced one of these two instruments for more than 1 year; “No” the piano or guitar practicing column refers to a blind has practiced one of these two instruments for less than 1 year or has never practiced them.

Early visual deprivation may lead to both abnormal and plastic changes in the visual and other systems of the brain [34], [35]. Although some previous MRI studies have revealed the brain structural and functional changes in early blindness [36]–[42], no one has yet investigated the topological alterations of the brain anatomical network due to early visual deprivation. In the present study, we employed deterministic DTT to infer the existence/absence of network connections [13]. The entire cerebrum was parcellated into 90 regions (see Materials and Methods, Table 2) using the AAL template to define the network nodes. Then we applied graph theory approaches to examine the topological properties of the network constructed for each subject and performed statistical analyses to explore the differences between early blind and normally sighted subjects in the topological properties of the network.

**Table 2:** Cortical and subcortical regions defined in Automated Anatomical Labeling template image in standard stereotaxic space.

Region Name	Abbreviation	Region Name	Abbreviation
Superior frontal gyrus, dorsolateral	SFGdor	Superior parietal gyrus	SPG
Superior frontal gyrus, orbital	SFGorb	Paracentral lobule	PCL
Superior frontal gyrus, medial	SFGmed	Postcentral gyrus	PoCG
Frontal gyrus, medial orbital	FGmedorb	Inferior parietal gyrus	IPG
Middle frontal gyrus	MFG	Supramarginal gyrus	SMG
Middle frontal gyrus, orbital	MFGorb	Angular gyrus	ANG
Inferior frontal gyrus, opercular	IFGoper	Precuneus	PCNU
Inferior frontal gyrus, triangular	IFGtri	Posterior cingulate gyrus	PCC
Inferior frontal gyrus, orbital	IFGorb		
Gyrus rectus	REG	Insula	INS
Anterior cingulate gyrus	ACC	Thalamus	THA
Olfactory cortex	OLF		
		Superior temporal gyrus	STG
Precentral gyrus	PreCG	Superior temporal gyrus, temporal pole	STGp
Supplementary motor area	SMA	Middle temporal gyrus	MTG
Rolandic operculum	ROL	Middle temporal gyrus, temporal pole	MTGp
Median- and para-cingulate gyrus	MCC	Inferior temporal gyrus	ITG
		Heschl gyrus	HES
Calcarine fissure and surrounding cortex	CAL	Hippocampus	HIP
Cuneus	CUN	Parahippocampal gyrus	PHIP
Lingual gyrus	LING	Amygdala	AMYG
Superior occipital gyrus	SOG		
Middle occipital gyrus	MOG	Caudate nucleus	CAU
Inferior occipital gyrus	IOG	Lenticular nucleus, putamen	PUT
Fusiform gyrus	FG	Lenticular nucleus, pallidum	PAL

Note: The abbreviations listed are those used in this paper, which differ slightly from the original abbreviations by Tzourio-Mazoyer et al. (2002).

## Results

To construct the binary anatomical network for each subject, we first partitioned the cerebrum into 90 regions to define the nodes. Then we performed whole-brain fiber tracking based on the DTI data, and if at least three fibers had their end-points in regions *u* and *v*, we connected the two nodes, *u* and *v*, with an edge.

### Altered topological properties of anatomical networks in early blindness

Based on the constructed network, we calculated the topological properties of the global network (see Materials and Methods) for each subject and show the mean values of these properties for the normal control (NC) and early blind (EB) groups in Table 3. Both groups exhibit efficient small-world properties and have almost identical path lengths (λ ≈ 1) but are more locally clustered (γ > 1) compared with the matched random networks. Two-sample t-tests indicated that the anatomical network for early blindness had a significantly increased characteristic path length (*L_p_*) (*p* = 0.02), a reduced global efficiency (*E_glob_*) (*p* = 0.01) and a decreased degree of connectivity (K*_p_*) (*p* = 0.008).

**Table 3:** The mean values of the topological properties of the networks for the NC and EB groups.

	γ	γ	σ	K_p_ *	L_p_ *	C_p_	E_glob_ *	E_loc_
NC	1.7307	1.0810	1.5991	14.2523	2.1722	0.4974	0.5264	0.7429
EB	1.8310	1.0860	1.6846	13.1137	2.2414	0.4987	0.5116	0.7420

* Significant group differences at p<0.05.

### Hub regions in anatomical network for each group

**Figure 1:**
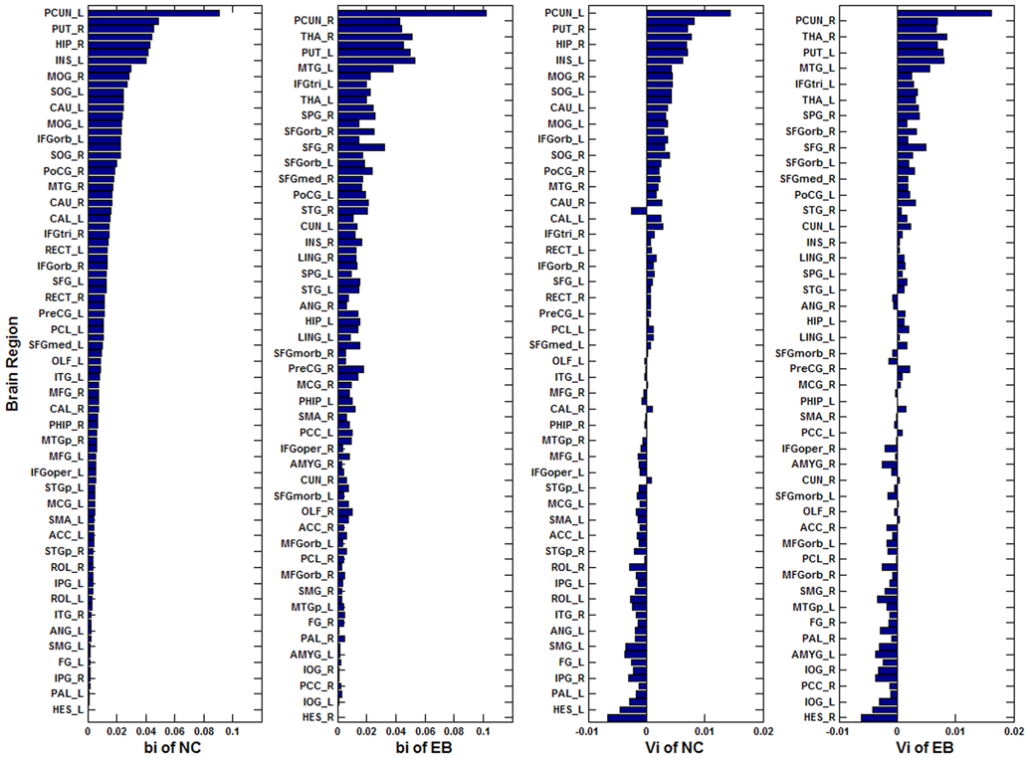
Comparison between two groups with respect to the b_i_ and V_i_ for each brain region. The length of the bar indicates the mean values sorted by the mean b_i_ of the NC group in descending order. Left two columns compare the b_i_ values of the control with those of the early blind. Right two columns make the same comparison for the V_i_ values.

First, we calculated the normalized betweenness centrality, *b_i_* and vulnerability *V_i_* for each node of each subject's anatomical network. Then we calculated the mean *b_i_* and *V_i_* of each node by averaging across subjects for each group. Figure 1 shows all 90 nodes sorted by the mean *b_i_* of the normal control group in descending order. To identify the hub regions, we examined the *b_i_* of each node in both networks (see Materials and Methods). For the control group, the identified hub nodes (19 total, Table 4) included 10 regions of the association cortex, 5 regions of subcortical structures, 3 paralimbic regions and 1 limbic region. The hubs for the early blind group included 7 regions of the association cortex, 5 regions of subcortical structures, 1 limbic region, 2 paralimbic regions and 1 region of primary cortex for a total of 16 regions (Table 5). The hubs that we located were predominantly in regions of heteromodal or unimodal association cortex which receive convergent inputs from multiple cortical regions [43].

**Table 4:** Hub regions of the NC group.

Regions	Class	*b_i_*	*k_i_*
Left precuneus	Association	0.0907	34
Right precuneus	Association	0.0487	27
Right putamen	Subcortical	0.0455	25
Right thalamus	Subcortical	0.0444	24
Right hippocampus	Limbic	0.0429	24
Left putamen	Subcortical	0.0418	26
Left insula	Paralimbic	0.0404	24
Left middle temporal gyrus	Association	0.0296	22
Right middle occipital gyrus	Association	0.0285	23
**Left inferior fronto-triangular gyrus**	Association	0.0272	21
Left superior occipital gyrus	Association	0.0249	23
**Left thalamus**	Subcortical	0.0247	19
Left caudate nucleus	Subcortical	0.0247	19
Right superior parietal gyrus	Association	0.0239	19
**Left middle occipital gyrus**	Association	0.0235	22
Right superior fronto-orbital gyrus	Paralimbic	0.0234	19
**Left inferior fronto-orbital gyrus**	Paralimbic	0.0229	20
Right superior frontal gyrus	Association	0.0226	19
**Right superior occipital gyrus**	Association	0.0223	22

**Table 5:** Hub regions of the EB group.

Regions	Class	*b_i_*	*k_i_*
Left precuneus	Association	0.102	32
Left insula	Paralimbic	0.0529	25
Right thalamus	Subcortical	0.0515	22
Left putamen	Subcortical	0.05	24
Right hippocampus	Limbic	0.0451	20
Right putamen	Subcortical	0.0443	23
Right precuneus	Association	0.0424	24
Left middle temporal gyrus	Association	0.0382	22
Right superior frontal gyrus	Association	0.0325	20
Right superior parietal gyrus	Association	0.0259	17
Right superior fronto-orbital gyrus	Paralimbic	0.0254	17
Left caudate nucleus	Subcortical	0.0248	18
**Right postcentral gyrus**	Primary	0.0237	16
Right middle occipital gyrus	Association	0.0227	18
Left superior occipital gyrus	Association	0.0225	18
**Right caudate nucleus**	Subcortical	0.0214	16

Note: The regions in bold are the different hubs between the NC and EB groups.

### Distribution of the altered regions in early blindness

**Figure 2:**
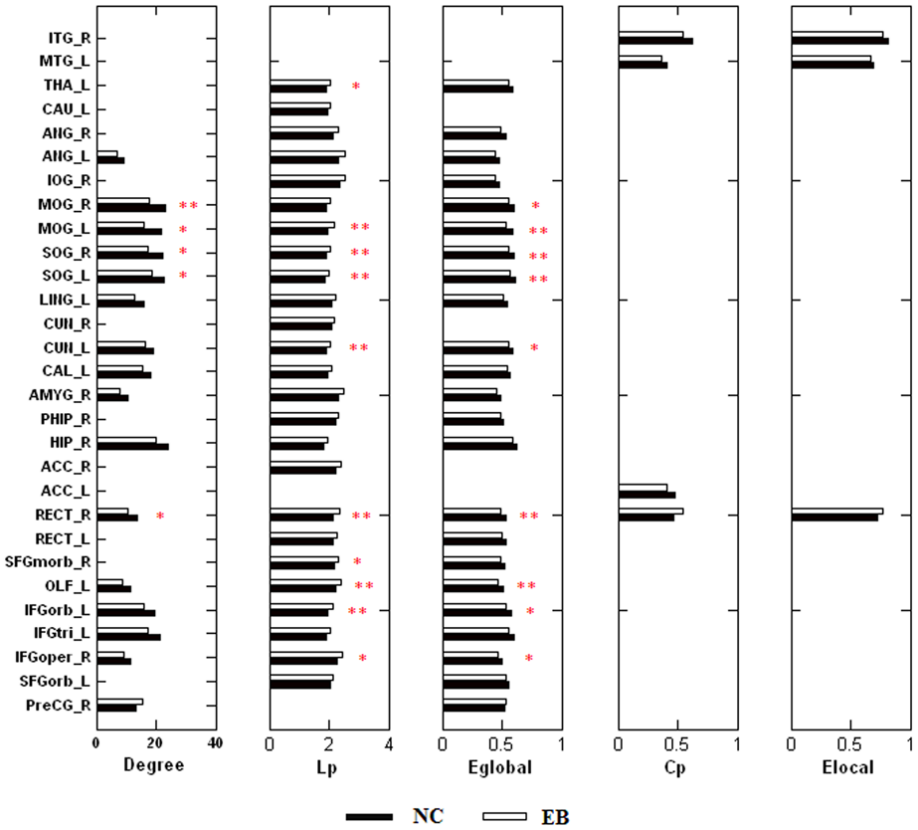
Distribution of regions with significantly altered nodal properties in early blind subjects. The length of the bar indicates the mean values of the topological measurements (Degree: *K_i_*; Lp: *L_i_*; Eglobal: *E_i_glob_*; Cp: *C_i_*; Elocal: *E_i_loc_*) of each altered brain region. Black bars represent normal group, and open bars represent early blind group. (Unstarred bars: significance with *p*<0.05; *: *p*<0.01; **: *p*<0.005).

**Figure 3:**
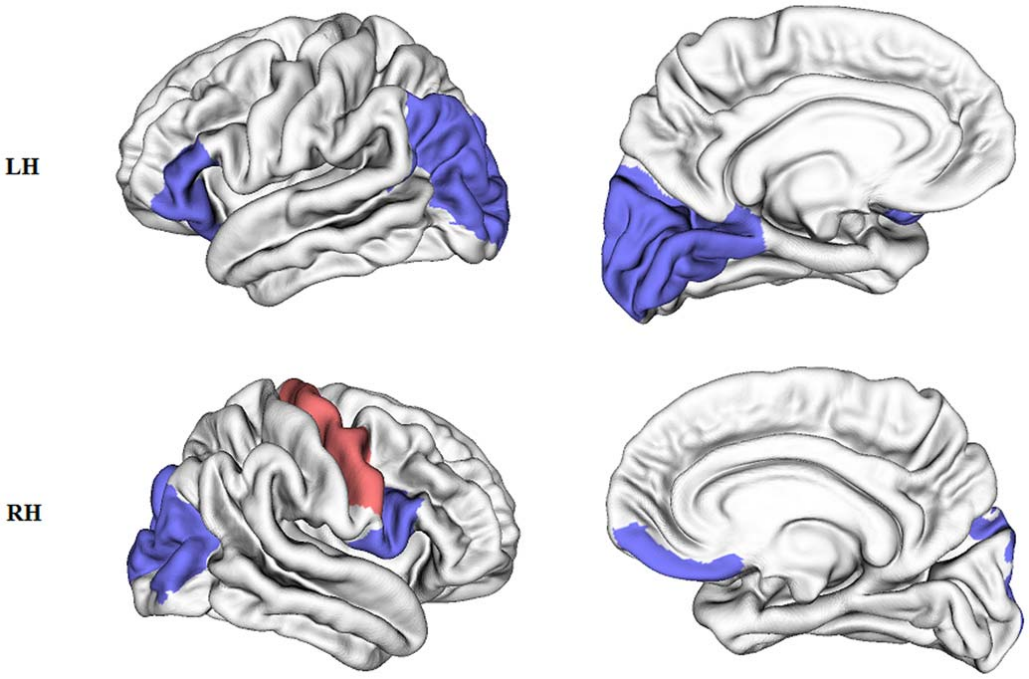
Cortical regions with altered topological properties in early blind subjects (*p*<0.05). Blue: regions with decreased *K_i_* and *E_i_glob_* and increased *L_i_* in the early blind; Red: regions with increased *K_i_* and *E_i_glob_* in the early blind; Top: left hemisphere (LH); Bottom: right hemisphere (RH).

Since the global properties, *L_p_*, *E_glob_* and K*_p_* of the anatomical network were significantly altered in the early blind group, we further investigated the distribution of the regions which showed significant differences in these topological properties. For each brain region, we used a two-sample two-tailed t-test to detect statistical differences in the nodal properties between groups, and selected the statistical thresholds at three different *p* values (0.05, 0.01 and 0.005) (Figure 2). The *L_i_*, *E_i_glob_* and K*_i_* were significantly altered in many brain regions, especially in the inferior frontal and occipital lobes (Figure 2, 3).

Although the local properties, such as C*_p_* and E*_loc_* of the global anatomical network were not significantly altered in early blind subjects, we performed statistical comparison of C*_i_* and E*_i_loc_* for each node to reveal the local changes of these two properties. The C*_i_* and E*_i_loc_* were altered in several regions with a statistical threshold at *p* = 0.05, such as gyrus rectus, middle and inferior temporal gyrus (Figure 2).

### Comparisons of backbone networks between groups

**Figure 4:**
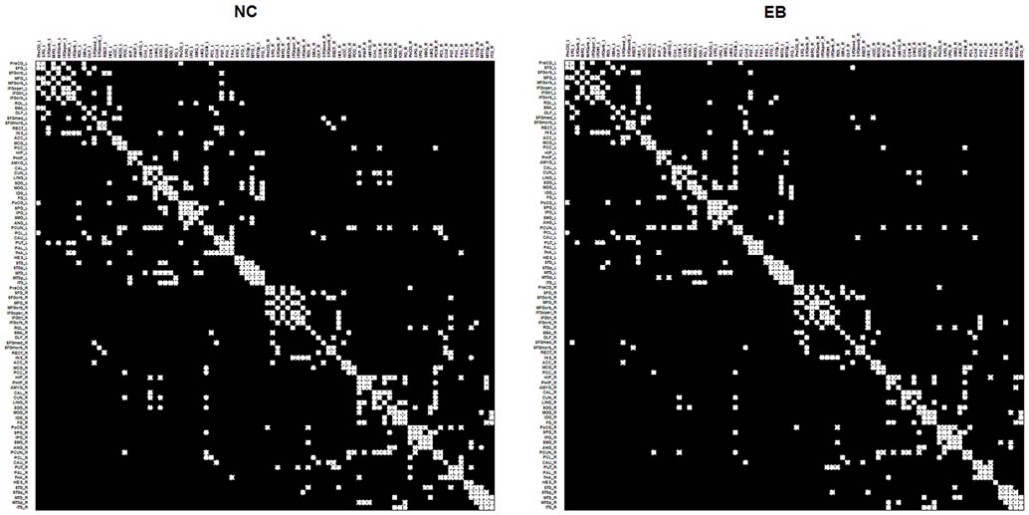
Backbone networks for the NC and EB groups.

We identified the population-based backbone network of each group separately. Figure 4 reveals that the control and early blind groups have similar connection patterns in 90 regions, but that the early blind group has a sparser matrix than the control group (sparsity of NC: 0.0649; sparsity of EB: 0.0562, reduced 13.4%). This finding suggests fewer anatomical connections in the early blind group at the same threshold. This is also consistent with the above result that showed a significantly reduced degree of connectivity in the early blind.

### White matter changes in early blindness

**Figure 5:**
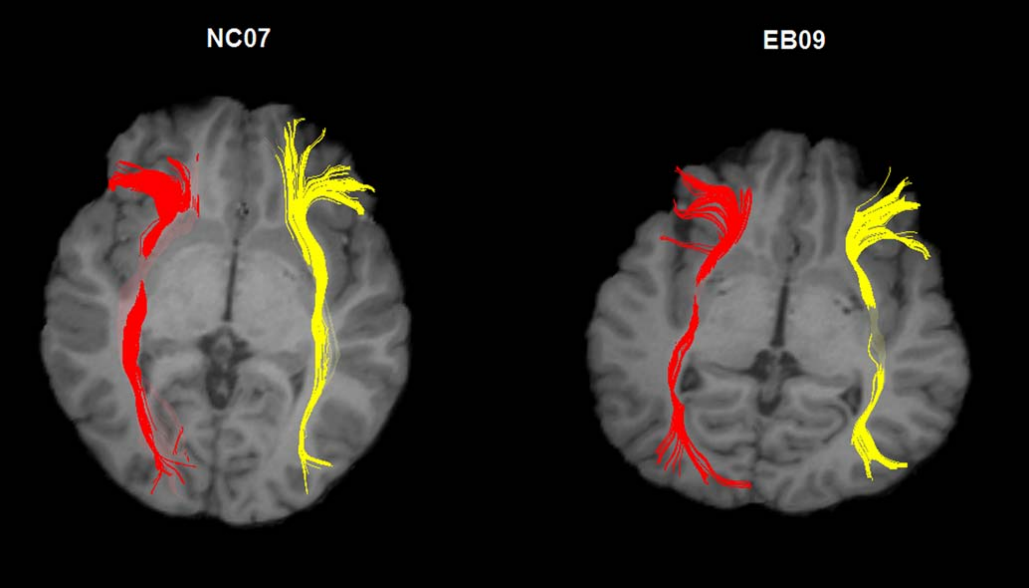
Representation of the reconstructed IFO fasciculi for a normal and blind subject. The tracked fibers are overlaied on the individual's anatomical image (T1-weighted). Left: normal subject NC07; right: blind subject EB09.

**Figure 6:**
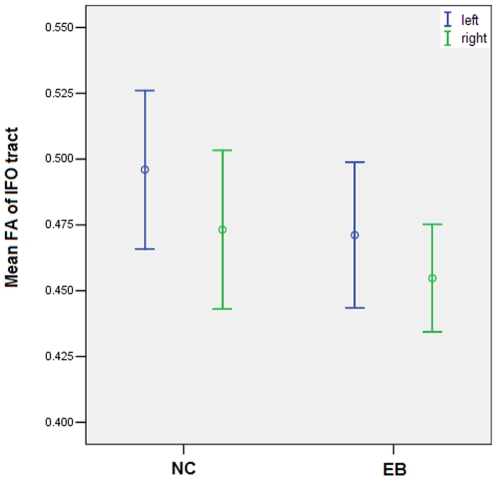
Mean FA of bilateral IFO fasciculi for the NC and EB groups. Open circles: mean values of the FA; error bars: standard deviations of the FA.

Through statistical comparison of nodal properties, we revealed most of altered brain regions were located in the inferior frontal and occipital lobe in the anatomical network, to further investigate whether these changes were related to the alterations of the integrity of the white matter tracts connecting these two regions, we reconstructed the long-distance anatomical connections between the inferior frontal lobe and the occipital lobe: the inferior fronto-occipital (IFO) fasciculus. For each subject, the bilateral IFO fasciculi can be well reconstructed (Figure 5). Through the statistical analysis of the mean fractional anisotropy (FA) in IFO fasciculi between groups, we found that the early blind subjects have significantly lower FA values than the sighted subjects (*p* = 0.002) (Figure 6). The analysis results also revealed there was a significant main effect of hemisphere (*p* = 0.005) and no significant group-by-hemisphere interaction (*p*>0.1).

## Discussion

The present study demonstrated changes in the anatomical network in early blindness using DTT for the first time. Compared with the normal controls, the early blind subjects showed disrupted global anatomical connection patterns, such as a lower degree of connectivity, a longer characteristic path length and a lower global efficiency. The brain regions with most significant topological changes were mainly located in the visual cortex, a finding which is consistent with previous MRI studies of the early blind [36]–[41], [44]. Therefore, our study suggests that investigating early blindness from the perspective of anatomical networks using DTI may be helpful for understanding of the brain's re-organization due to early visual deprivation.

### Small-world anatomical networks

In this study, we revealed the small-world properties of the anatomical networks in both the controls and the early blind subjects. These findings are consistent with previous studies, which have revealed the small-world topologies of the human brain using various techniques, such as electroencephalography [45], [46], magnetoencephalography [3], functional MRI [5], [12], structural MRI [8] and diffusion MRI [9], [11], [13]. Small-world networks have high clustering coefficients and short characteristic path lengths and are thus good compromises between regular and random networks [33]. Small-world topologies can support both segregated/specialized and distributed/integrated information processing. Moreover, small-world networks are economical, tending to minimize wiring costs while supporting a high level of dynamical complexity [47]. Dynamic systems with small-world properties seem to confer resilience against pathological attack and display enhanced signal-propagation speed, computational power, and synchronizability [2], [6], [10]. Our findings provided support for the perspective that the human brain has evolved into a highly complex but efficient neural system [2], [48].

### Altered anatomical networks in early blindness

The human brain is a large, dynamic system with an optimal balance between local specialization and global integration. Although small-world properties were present for both the normal control and early blind groups, the topological architectures of the anatomical networks were significantly altered in the early blind subjects.

#### Altered topological properties of global networks in early blindness

Statistical analysis of the topological properties at the global level revealed that the early blind showed a significantly decreased degree of connectivity, a decreased global efficiency and an increased characteristic path length compared with controls. A decreased degree implies that the connections in the network are relatively sparse. The lower global efficiency and longer characteristic path length indicate that information transfer and interactions between brain regions are slower and less efficient. Considering the interactions between these three topological properties, less degree will increase the overall travel distance. In turn, as global efficiency is conceptual identical to characteristic path length, global efficiency will decrease. However, no group difference was found in normalized lambda (λ), which includes a correction for a reduced degree in the network. The non-effect on lambda may suggest that the level of efficiency itself is not reduced, but that the main effect is that a number of (vital) cortical-cortical connections are missing/reduced in the early blind. In future studies we will investigate the “relative” network properties by constraining each network with the same number of nodes and connections to further investigate the alteration of network organization in the blind subjects.

For early blind, reduced cortical-cortical connections could be related to abnormal development of the cerebral white matter due to early visual deprivation. These results are consistent with previous studies which suggested that the loss of visual experience can alter the structural organization in both the gray matter and the white matter during a critical period of neurodevelopment [35], [49]–[51]. Recently, a structural MRI study found that the early blind had decreased gray matter volume in the early visual areas, accompanied by atrophy of the optic chiasm and the optic radiation [37]. It also corresponded with the findings of a previous DTI study on the same population, showing reduced visual network integrity in the early blind [39] and the above results of reduced FA in the IFO fasciculus.

#### Distribution of brain regions with altered topological properties in early blindness

Since we found changes in the global topological properties, such as *L_p_*, *E_glob_* and K*_p_* between the two groups, we further investigated the distributions of the regions which showed significant differences in these nodal properties. Figures 2 and 3 indicate that most of regions with disrupted topological attributes were located in the occipital lobe.

The occipital lobe contains the visual processing center of the mammalian brain. In this study, we found that the superior and middle occipital gyrus, and the cuneus were the most significantly altered regions in the occipital lobe of the early blind, with decreased degree and global efficiency and increased characteristic path length. These changes suggest that these regions have reduced connections with other brain regions compared with normally sighted subjects. Be worth mentioning, in the present study the connections of the anatomical network are related to the number of fibers to be traced from one region to another, which is largely dependent on the FA values of the white matter tracts. Both previous [39] and current DTI studies with the same dataset have revealed reduced FA in the visual tracts, such as the optic radiation and IFO fasciculus of the early blind, Therefore, this will lead to less traceable fibers and less connectivity between regions at a group level concerning the selected threshold of 3 fibers.

Moreover, we found that the precentral gyrus, which contains the motor area, was the only region with increased degree and global efficiency in the early blind, when analyzed at a loose statistical threshold (*p* = 0.05). This finding indicated that the precentral gyrus had more or strengthened connections with other brain regions, therefore predicting better performance in information transfer and interaction than in sighted subjects. This finding could suggest experience-dependent compensatory plasticity [52], [53]. In the absence of visual experience, early blind subjects need more practice to perform the same routine activities of the sighted subjects, and they engage in much finer finger movements, such as tactile exploration of objects and Braille reading (all the early blind subjects), massage (12/17 early blind subjects) and piano or guitar practicing (7/17 early blind subjects). The enhanced motor activity in early blind subjects may increase the number, diameter and in particular myelination of the relevant axons, especially during the critical early period of neurodevelopment. This result is consistent with a previous study which revealed plasticity of the corticospinal tract in the early blind [40]. It is also supported by a VBM study that found significant white matter increases in the sensory-motor system of the early blind [37].

Compared with the altered regions in *L_i_*, *E_i_glob_* and K*_i_*, the regions with altered C*_i_* and E*_i_loc_* were different and much less. For early blind, only one region with increased local efficiency and clustering coefficient was the right gyrus rectus, and this region is also changed with increased characteristic path length and reduced global efficiency. It suggests increased short connections and decreased long connections of this region. Additionally, the regions with decreased local efficiency and clustering coefficient were located in the middle and inferior temporal gyrus, which suggests the short connections within the neighbors of these regions are much reduced.

#### Alteration of hub regions in anatomical networks of early blind

The measurement of the betweenness centrality and the analysis of the vulnerability allowed us to identify the most critical anatomical nodes in the brain, revealing quantitative information about the global damage that could be caused by a hypothetical failure of these nodes. In this study, we used the level of betweenness centrality to identify the hub regions of the anatomical networks [8], [13]. Most of the hub regions (14 regions), such as the precuneus, putamen, hippocampus, middle temporal gyrus, superior parietal gyrus and superior frontal gyrus, were the same in both the control and early blind groups (Table 4 and 5). We found that most of the hub regions that we identified were located in the association cortex, which plays a central role in receiving convergent inputs from multiple cortical regions [43]. These findings are in accordance with several previous studies in which other researchers have identified these association cortex regions as critical nodes in both structural and functional brain networks in human [5], [8], [11], [13], [15] and nonhuman primates [2], [54], [55]. Furthermore, some regions of subcortical structures, such as the thalamus, putamen and caudate nucleus, and some limbic and paralimibic regions, such as the hippocampus and insula, were also revealed as the hub regions of the anatomical networks, which are consistent with the findings of a previous DTI study [11].

However, compared with the normal control group, the early blind group lacked five hub regions, i.e. left middle occipital gyrus, right superior occipital gyrus, left inferior fronto-orbital gyrus, left inferior fronto-triangular gyrus and left thalamus. This suggests a decreased importance of the frontal and occipital regions or their connections in the early blind. In order to examine the relationship between the disruption of local white matter and the abnormal nodal properties, we reconstructed the long-distance anatomical connections (IFO fasciculus) which connect the inferior frontal lobe with the occipital lobe. By comparing the FA values of the IFO fasciculi between groups, we found that the early blind subjects had significantly lower FA values in the IFO fasciculi than did sighted subjects. This difference suggests a degree of disrupted integrity of the white matter tract. Previous DTI studies using the same dataset revealed disrupted integrity of the optic radiation [39] and plastic changes in the corticospinal tract [40] of the early blind. All these results suggest that local diffusion changes in the white matter can be revealed by the graph theoretical analysis of the anatomical network.

Two hub regions, the right postcentral gyrus and the right caudate nucleus, appeared in the early blind group, but not in the control group. This finding suggests that these two regions have important global roles in the early blind. The postcentral gyrus is known as the primary somatosensory cortex [56], [57]. As we discussed above, early blind subjects are likely to engage in more frequent tactile exploration of objects and in Braille reading than are sighted subjects. This may enhance the somatosensory ability of the early blind. Therefore, an increased importance of the postcentral gyrus in the anatomical network may suggest an experience-dependent compensatory plasticity due to early visual deprivation [34], [52], [53]. The other hub region, the caudate nucleus, together with the putamen, composes the dorsal striatum, which is the gateway to the basal ganglia. The dorsal striatum receives convergent excitatory afferents from the cortex and the thalamus and is the origin of the basal ganglia circuits involved in motor control [58], [59]. Although no study has investigated structural or functional changes in the caudate nucleus in the early blind, we speculate that the increased betweenness centrality of the caudate nucleus in the anatomical network demonstrates the functional importance of motor control for the early blind as a result of visual loss. This may also suggest striatal plasticity, which has been reported in previous studies [60], [61].

#### Functional relevance

Previous neuroimaging studies have focused on functional reorganization following blindness. The visual cortex of the early blind was activated during performing different tasks, such as language processing [62]–[64], tactile discrimination [65]–[67], auditory stimuli [68], [69] or when working on higher cognitive tasks [63]. The recruitment of regions that typically respond to visual stimuli has been attributed to altered connectivity between primary visual areas and brain regions subserving other sensory modalities [70]. Recently, functional MRI studies have demonstrated a general loss of functional connectivity in the early blind [36], [41], which has a correspondence with the reduced cortical-cortical connections revealed in this study. It is also suggested the resting-state functional connectivity is constrained by the anatomical structure of the human cerebral cortex [71]. In future studies, we will investigate the functional networks of the early blind to further address the relationship between altered structural and functional properties of the human brain network.

### Methodological issues

The most essential elements of a network are the nodes and edges. The definition of the nodes and edges has a great effect on the constructed network. Therefore, we need to address some methodological issues about how we carried out the network construction:

First, because this is an exploratory study, we applied the AAL template and automatic registration by the software of statistical parametric mapping (SPM, http://www.fil.ion.ucl.ac.uk/spm) to define the nodes for each subject's network. Previous researchers have proposed different parcellation strategies for the cerebral cortex [5], [8], [12], [15]. And one fMRI study, by employing different parcellation schemes to construct the functional networks, suggested that the topological organization of the brain network can be affected by the brain parcellation atlases [72]. Recently, Hagmann et al. have proposed a more fine grained representation which is defined at a voxel population level rather than a regional level to partition the cerebral cortex into thousands of regions [15]. In future studies, such advanced parcellation method should be employed to investigate the brain network in specific/diseased populations, in order to localize the alterations of the topological organization more accurately. Another related concern is the registration between the template and individual images. An automatic registration technique by SPM can not guarantee an exact match of every anatomical location across the subjects, so it may cause the network nodes to have slight location errors [73]. In future studies, we will further confirm our results by applying different parcellation schemes and registration techniques.

Second, in this study, we employed deterministic DTT to define the edges of the anatomical network. However, the “fiber crossing” problem is a limitation of deterministic tractography algorithms, because the tracking always stops when it reaches fiber crossing regions with low factional anisotropy values [28]. This will result in the loss of some existing fibers, and hence some edges of the network. Recent studies have proposed advanced imaging techniques, such as DSI [74], [75] or high angular resolution diffusion imaging (HARDI) with Q-ball reconstruction of multiple fiber orientations [76], [77], for solving the “fiber crossing” problem. Another limitation of deterministic tractography, especially for long-distance fiber bundles, is erroneous tracking results due to noise and resolution limitations [28]. To solve this issue, several researchers have used probabilistic fiber tracking algorithms [30], [78], [79]. By modeling a probability distribution of the fiber orientations within a voxel, these statistical methods can identify fiber connections missed by deterministic tracking approaches. However, the number of gradient directions in our diffusion dataset is not sufficient to accurately estimate the probability density function of the fiber orientations. Therefore, future studies with more advanced diffusion imaging techniques or tractography methods could yield a more complete and accurate anatomical network for each subject.

Another issue about the choice of a binary or weighted network needs addressing. For a weighted network, a challenge is to decide on the most representative measure of structural connectivity. Several candidate measures, such as fiber numbers, mean fiber length, fiber density, and mean fraction anisotropy can be selected as the connectivity measure [9], [31], [80]. But the physiological meaning of these measures is unclear. It is also hard to validate which measure describes the information transfer of neural signals most accurately. In this work, we constructed the binary network by just taking into consideration the existence/absence of regional connections. However, a weighted network with a proper connectivity measure may better reflect network changes in specific/diseased populations.

## Materials and Methods

### Subjects

This study included 17 early blind subjects (10 males, 7 females; mean age 22 years, range 16–29 years) and 17 age and gender-matched normally sighted controls (10 males, 7 females; mean age 23 years, range 19–28 years). All blind subjects were recruited from the Special Education College of Beijing Union University. Onset of blindness was at birth or within the first year of life for all blind individuals. The demographic data of the early blind are shown in Table 1. All participants were right-handed, based on the Edinburgh handedness inventory [81]. Each participant provided written informed consent before their MRI examination, and the Medical Research Ethics Committee of Xuanwu Hospital of Capital Medical University approved the study. We used this dataset in one of our previous studies on early blindness, which focused on the diffusion changes of the white matter in early blind subjects [39].

### Data acquisition

DTI was performed with a 3.0 T Siemens Trio MR system using a standard head coil. Head motion was minimized with restraining foam pads provided by the manufacturer. Diffusion weighted images were acquired employing a single-shot echo planar imaging (EPI) sequence in alignment with the anterior-posterior commissural plane. The Integral Parallel Acquisition Technique (iPAT) was used with an acceleration factor of 2. Acquisition time and image distortion from susceptibility artifacts can be reduced by the iPAT method. Diffusion sensitizing gradients were applied along 12 non-linear directions (b = 1000 s/mm^2^) together with an acquisition without diffusion weighting (b = 0 s/mm^2^). The imaging parameters were 45 continuous axial slices with a slice thickness of 3 mm and no gap, field of view  = 256 mm×256 mm, repetition time/echo time  = 6000/87 ms, acquisition matrix  = 128×128. The reconstruction matrix was 256×256, resulting in an in-plane resolution of 1 mm×1 mm. For each participant, a sagittal T1-weighted 3D image was also collected using a magnetization prepared rapid gradient echo (MP-RAGE) sequence. The imaging parameters for this were a field of view of 22 cm, repetition time/echo time  = 24/6 ms, flip angle  = 35° and voxel-dimensions of 1 mm×1 mm×1 mm.

### Data preprocessing

Image distortions and motion artifacts in the DTI dataset were corrected by applying affine alignment of each diffusion-weighted image to the b = 0 image, using FMRIB's Diffusion Toolbox (FSL, version 3.3; www.fmrib.ox.ac.uk/fsl). After this process, the diffusion tensor elements were estimated by solving the Stejskal and Tanner equation [23], [24], and then the reconstructed tensor matrix was diagonalized to obtain three eigenvalues (λ_1_, λ_2_, λ_3_) and eigenvectors. The FA of each voxel was calculated according to the following formula:
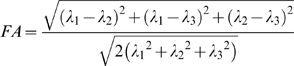
DTT was implemented with DTIstudio, Version 2.40 software (H. Jiang, S. Mori; Johns Hopkins University), by using the “fiber assignment by continuous tracking” method [82]. All tracts in the dataset were computed by seeding each voxel with an FA greater than 0.2. Tractography was terminated if it turned an angle greater than 50 degrees or reached a voxel with an FA less than 0.2 [83].

### Construction of anatomical networks

We constructed an anatomical network for each subject based on fiber connectivity from DTT with the main procedures as follows: First, we automatically segmented the cerebrum into 90 cortical and subcortical regions (45 for each hemisphere, regions of cerebellum were excluded, see Table 2) using the AAL template (see Gong et al. 2009 for details), which has been used in several previous studies [5], [11], [12], [17], [84]–[86]. Each region represents a node of the network. Second, we used the streamline fiber tracking method to obtain a map of all the fibers in the brain. If at least three fibers had their end-points in regions *u* and *v*, we connected the two nodes, *u* and *v*, with an edge. A threshold of three fibers ensured that the average size of the largest connected component of the network was 90 across all subjects. The number of fibers between regions only indicated the existence/absence of an edge. In this way we constructed a binarized anatomical network for each subject and represented it in a symmetric 90×90 matrix.

### Graph theoretical analysis

We investigated the topological properties of the anatomical network at the global and regional (nodal) levels, quantifying the global network architecture in terms of the small-worldness, clustering coefficient (*C_p_*), characteristic path length (*L_p_*), local efficiency (*E_loc_*) and global efficiency (*E_glob_*) of the whole brain network. We described the regional properties in terms of degree (K*_i_*), clustering coefficient (*C_i_*), shortest path length (*L_i_*), local efficiency (*E_i_loc_*), global efficiency (*E_i_glob_*), betweenness centrality (*B_i_*) and vulnerability (*V_i_*) of the node *i*. Based on the constructed anatomical network of each subject, we looked for significant differences in global and nodal properties between the normally sighted and early blind groups.

### Topological properties of the network

Because we constructed an N×N (N = 90) binary graph, consisting of the nodes (brain regions) and undirected edges between nodes for each subject, here we only provide brief, formal definitions of each of the network properties used in this study.

#### Degree, clustering coefficient, shortest path length, and small-worldness

The degree K*_i_* of a node *i* is defined as the number of connections to that node. Highly connected nodes have a large degree. The degree K*_p_* of a graph is the average of the degrees of all nodes in the graph:
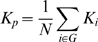
which is a measure that is used to evaluate the degree of sparsity of a network.

The clustering coefficient *C_i_* of a node *i* is defined as the number of existing connections among the node's neighbors divided by all their possible connections:
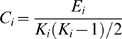
where *E_i_* is the number of existing connections among the node's neighbors [33]. The clustering coefficient of a network is the average of the clustering coefficient of all nodes:
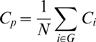
in which *C_p_* quantifies the extent of local cliquishness or local efficiency of information transfer of a network [33], [87].

The mean shortest path length *L_i_* of a node *i* is: 
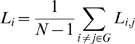
in which *L_i,j_* is the smallest number of edges that must be traversed to make a connection between node *i* and node *j*. The characteristic path length of a network is the average of the shortest path length between the nodes: 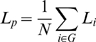
in which *L_p_* quantifies the ability to propagate parallel information or global efficiency (in terms of 1/*L_p_*) of a network [87].

The concept of “small-world” was originally proposed by Watts and Strogatz [33]. Compared with random networks, small-world networks have similar characteristic path lengths but higher clustering coefficients, that is 
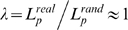


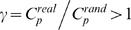
where the 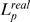 and 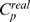 are the characteristic path length and clustering coefficient of the real network, the 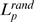 and 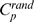 are the mean characteristic path length and clustering coefficient of 100 matched random networks that preserve the same number of nodes, edges, and degree distribution as the real network [2], [88]. These two conditions can be summarized into a scalar quantitative measurement, small-worldness, 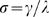
which is typically greater than 1 for small-world networks [5], [8], [12], [89].

#### Network efficiency

Small-world networks are very efficient in terms of global and local communication. They have high global efficiency *E_glob_* and local efficiency *E_loc_* [17], [87].

The global efficiency *E_i_glob_* of a node *i* is defined as: 
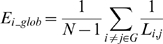
The global efficiency of the network *E_glob_*, a measure of the global efficiency of the parallel information transfer in the network, is defined by the inverse of the “harmonic mean” of the shortest path length between pairs of nodes: 
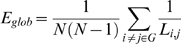



The local efficiency *E_i_loc_* of a node *i* can be calculated as:
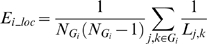
where the subgraph *G_i_* is the set of nodes that are the direct neighbors of the node *i*. This measure reveals how much the network is fault tolerant, showing how efficient the communication is among the first neighbors of the node *i* when it is removed. The mean local efficiency of a graph is defined as:
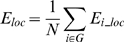
which is the mean of the local efficiency of all the nodes in the graph.

#### Betweenness centrality and vulnerability

Betweenness centrality is widely used to identify the most central nodes in a network, which are associated to those nodes that act as bridges between the other nodes. The betweenness *B_i_* of a node *i* is defined as the number of shortest paths between pairs of other nodes that pass through the node *i* [90], [91]. The normalized betweenness *b_i_* was then calculated as: 
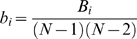
The nodes with the largest normalized betweenness values were considered to be pivotal nodes (i.e., hubs) in the network. Specifically, nodes were identified as hubs in the network if *b_i_* was greater than 1.5 times the average betweenness of the network (i.e., *b_i_*>1.5mean) [92].

The vulnerability analysis can quantitatively measure the damage on the network performance caused by the hypothetical failure of its elements [93]. The vulnerability *V_i_* of a node *i* is defined as the changes in global efficiency of the network when node *i* and its edges are removed. The vulnerability can be calculated as: 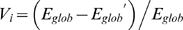
where *E_glob_'* is the global efficiency of the network after removing node *i* and its edges. Vulnerability is also an important measure as betweenness centrality for characterizing the influence of nodes in a network. Group differences in *B_i_* and *V_i_* reflect the effects of the disease on the global roles of regions in the anatomical network.

### Backbone network of each group

The backbone network across the population of each group was calculated using Gong's method [13]. Non-parametric one-tailed sign tests (*p* = 10^−4^, uncorrected) were applied to identify the consistent anatomical connections in each group.

### Reconstruction of inferior fronto-occipital fasciculus

Based on anatomical knowledge of fiber projections, several studies have suggested tracking protocols for the IFO fasciculus [94]–[96]. According to the published tracking protocols, we identified the first ROI at the middle point between the posterior edge of the cingulun and the posterior edge of the parieto-occipital sulcus on a coronal slice [95]. For the second ROI, a coronal slice is selected at the anterior edge of the genu of corpus collasum and the entire hemisphere is delineated [95]. All ROIs were identified on the individual's FA-weighted color maps. Fiber bundles that passed through both ROIs were selected as belonging to the IFO fasciculus.

For each subject, we can reconstruct the bilateral IFO fasciculi successfully (Figure 5). After that, we calculated the mean FA for each fiber tract by averaging the FA values across the voxels that form the three-dimensional tracts derived from tractography. Then we compared the mean FA values of the bilateral IFO fasciculi between the normal control and early blind groups.

### Statistical analysis

Based on the binary network constructed for each subject, we performed statistical comparisons of the *L_p_*, *C_p_*, *E_glob_*, *E_loc_* and K*_p_* values of the global networks between groups using a two-sample two-tailed t-test with a threshold of *p* = 0.05. Furthermore, we investigated the distributions of the regions which showed significant differences in their nodal properties.

We first calculated the mean FA values of the IFO fasciculus for each hemisphere of each subject. A two-factor analysis of variance (ANOVA) (group as a between-subjects factor and hemisphere as a within-subject factor) was used to assess between-group differences in FA values. The tests were considered significant when the *p* values were less than 0.05 (two-tailed).
